# Array-CGH analysis in Rwandan patients presenting development delay/intellectual disability with multiple congenital anomalies

**DOI:** 10.1186/1471-2350-15-79

**Published:** 2014-07-12

**Authors:** Annette Uwineza, Jean-Hubert Caberg, Janvier Hitayezu, Anne Cecile Hellin, Mauricette Jamar, Vinciane Dideberg, Emmanuel K Rusingiza, Vincent Bours, Leon Mutesa

**Affiliations:** 1 Center for Medical Genetics, College of Medicine and Health Sciences, University of Rwanda, Huye, Rwanda; 2 Center for Human Genetics, Centre Hospitalier Universitaire Sart-Tilman, University of Liege, Liege, Belgium; 3 Department of Pediatric Cardiology, Kigali University Teaching Hospital, Kigali, Rwanda

**Keywords:** Developmental delay, Intellectual disability, Multiple congenital abnormalities, Array-CGH, Copy number variation, Rwandan patients

## Abstract

**Background:**

Array-CGH is considered as the first-tier investigation used to identify copy number variations. Right now, there is no available data about the genetic etiology of patients with development delay/intellectual disability and congenital malformation in East Africa.

**Methods:**

Array comparative genomic hybridization was performed in 50 Rwandan patients with development delay/intellectual disability and multiple congenital abnormalities, using the Agilent’s 180 K microarray platform.

**Results:**

Fourteen patients (28%) had a global development delay whereas 36 (72%) patients presented intellectual disability. All patients presented multiple congenital abnormalities. Clinically significant copy number variations were found in 13 patients (26%). Size of CNVs ranged from 0,9 Mb to 34 Mb. Six patients had CNVs associated with known syndromes, whereas 7 patients presented rare genomic imbalances.

**Conclusion:**

This study showed that CNVs are present in African population and show the importance to implement genetic testing in East-African countries.

## Background

Intellectual disability (ID) is described by significant limitations in both intellectual functioning and adaptive behaviour that begin before the age of 18 years. A diagnosis of intellectual disability is usually made when IQ testing reveals an IQ of less than 70, which means that often the diagnosis is not made until late childhood or early adulthood. However, most persons with intellectual disability are identified early in childhood on the basis of concern about developmental delays, which may include motor, cognitive, and speech delays [1,2]. Developmental delay (DD) is a subset of developmental disabilities defined as significant delay in two or more of the following developmental domains: gross/fine motor, speech/language, cognition, social/personal, and activities of daily living [3]. Intellectual disability can range from mild to profound and can be associated with other clinical findings as part of a syndrome or can occur as an isolated phenotype [4]. When ID and DD are associated with multiple congenital anomalies (MCA), chromosomal abnormalities are suggested as the most frequent cause [5].

The prevalence of intellectual disability occurs in 2–3% of the general population worldwide [6,7], however little is known at this time about genetic causes of DD/ID in low-income countries [8]. Right now, there is no available data on genetics of DD/ID and MCA in East Africa. In low-income countries, environmental factor such as malnutrition, infections, birth asphyxia, cultural deprivation, poor health and parental consanguinity play a key-leading role in the occurrence of ID and DD [7,9]; but the involvement of genetics diseases in the occurrence of these impairments cannot be ignored.

During the last five years, array comparative genomic hybridization (array-CGH) has revolutionized the diagnostic approach to children with unexplained DD/ID and congenital malformations and has become the first-tier investigation in such patients [10].

Here, we present the first Rwandan study of array-CGH application in a selected cohort of 50 children with DD/ID/MCA.

## Methods

### Patients

Fifty Rwandan patients who consulted the department of medical genetics of the University Teaching Hospitals of Rwanda (Kigali and Butare) form January 2010 to December 2012 were included in this study. The male/female ratio was 1.94. The mean age was 6.41 ± 5.8. Inclusion criteria were the presence of DD/ID associated with MCA.

After clinical evaluation by a clinical geneticist, patients received genetic routine evaluation including the FMR1 gene study (Fragile-X) and standard karyotype. Patients with trisomy (21, 13 and 18) were not included in the cohort. The karyotypes of 47 patients were normal but 3 patients had abnormal karyotypes. These abnormalities consisted of a supernumerary maker, a duplication 1p and a terminal deletion 10p.

The study was approved by the Rwandan National Ethics Committee (N°394/RNEC/2013). Signed informed consent forms and permission for publication of this report and any accompanying images were obtained from the parents or legal guardians of all patients.

### DNA extraction

DNA was extracted from peripheral blood leukocytes using the phenol/chloroform method and following manufacturer’s instructions. DNA extraction was performed at the Laboratory of Medical Genetics of the University of Rwanda, and then transferred to the Center for Human Genetics at Liege-Belgium in appropriate conditions.

### Array CGH analysis

Oligonucleotide array-CGH was performed in fifty patients using SurePrint G3 Human CGH Microarray ISCA 4x180K v2 (AMADID 031748; Agilent Technologies, Santa Clara, CA, USA). The 180 K kit (180,000 probes) has an overall median probe spacing of 13 kb. Analysis was performed according to the protocol provided by the supplier (Agilent Oligonucleotide Array-Based CGH for Genomic DNA Analysis, version 6.3). Arrays were scanned using a SureScan High Resolution Microarray Scanner (Agilent). Data were imported using the Feature Extraction V.9.5.3.1 software and results were analyzed using CytoGenomics Analysis software v2.5 (Agilent). The Aberration Detection Methods 2 algorithm (ADM2) was used to analyze data with a threshold of 6.0 and a moving average window of 0.2 Mb. Log 2 ratios under _0.4 and variations with less than four consecutive probes were excluded. Genomic positions were based on the UCSC February 2009 human reference sequence (hg19) (NCBI build 37 reference sequence assembly). Filtering of CNVs was carried out using the BENCHlab CNV software (Cartagenia, Leuven, Belgium). Gene informations were collected from available literature and different database as described before [11].

### Confirmatory analysis

Multiplex ligation-dependent probe amplification (MLPA) and Fluorescence in situ hybridization (FISH) were performed to confirm the results of array-CGH and the mode of inheritance. An additional file shows some MLPA results (see Additional file 1).

### Multiplex ligation-dependent probe amplification analysis

MLPA analysis was carried out according to the manufacturer's instructions (MRC Holland, Amsterdam, Netherlands), using the SALSA probe mix P036 and P070 Human Telomere and SALSA probe mix P245 microdeletion syndrome. Amplification products were analyzed using capillary electrophoresis on ABI PRISM 3100 Genetic Analyzer. The data obtained were analyzed using the Sequence Pilot software (JSI medical systems, Kippenheim, Germany).

### Fluorescence in situ hybridization analysis

FISH was performed using standard protocols with commercially available probes as previously described [11]. Chromosomal abnormalities detected by array-CGH were confirmed and visualized by metaphase FISH using corresponding BAC clones. When available, parental chromosomes were also analyzed by metaphase FISH to exclude inherited rearrangements.

## Results

Array-CGH revealed copy number variations (CNV) of clinical significance in 13 patients giving a diagnosis rate of about 26%. Twelve patients presented one chromosomal aberration, while two concomitants abnormalities (i.e. 1 duplication and 1 deletion) were detected in 1 patient (patient 36). The size of the CNVs ranged from 0,9 Mb to 34 Mb. Six patients had CNVs related to known syndromes including William-Beuren syndrome (microdeletion 7q23.11,OMIM 194050, patient 6), deletion 22q11.21 (OMIM 192430, patient 14 and 37), duplication 7q23.11 (OMIM 609757, patient 34), deletion 8p23.1 (patient 17) and deletion 17q21.31 (OMIM 610443: patient 45). Seven patients presented rare genomic imbalances: trisomy 18p (patient 1), deletion 6q16.1-q21 (patient 13), duplication 1p35.3-p31.3 (patient 16), deletion 8p23.1 (patient 17), deletion 7q34-q36.2 (patient 18), deletion 2q33.1-q33.3 (patient 20), deletion 10p15.3-p14 (patient 39) and microduplication 8q24.3 concomitant with microdeletion 16p13.3 (patient 36). Eight patients presented a *de* novo aberration (16%), one abnormality was maternally inherited. In four patients the mode of inheritance was not investigated since the parents’ DNA was not available (Table 1).

**Table 1:** Array-CGH results and clinical features of the 13 Rwandan patients with pathogenic CNVs

**Patient**	**Age**	**Gender**	**Array result**	**Size**	**Inheritance**	**Clinical features**
1	6 y	F	arr [hg19] 18p11.32p11.21 (108,760-14,241,744)x3; 18p11.21q11.2(15,345,079-18,270,513)x3	14 Mb	*de novo*	DD, moderate ID, facial dysmorphism, hypertelorism, AVSD with ASD, single transverse palmar crease.
6	11 y	M	arr [hg19] 7q11.23 (72,700,414-74,142,327)x1	1442 kb	*de novo*	Moderate ID, facial dysmorphism, friendly behaviour, Mitral valve prolapse.
13	6 y	M	arr [hg19] 6q16.1q21 (93,818,221-108,052,559)x1	14 Mb	Unknown	Absence of speech with severe ID, facial dysmorphism, ear abnormalities, microcephaly, bilateral cryptorchidism and autistic-like behavior and underweighted.
14	15 y	M	arr [hg19] 22q11.21 (18,706,001-21,464,119)x1	2758 kb	*de novo*	Mild ID, hypotonia at birth, facial dysmorphism, hypernasal speech, a short stature.
16	6 y	M	arr [hg19] 1p35.3p31.3 (29,531,861-63,886,221)x3	34 Mb	*de novo*	Moderate ID, anxiety and hearing impairment. Facial dysmorphism, clinodactyly.
17	9 y	M	arr [hg19] 8p23.1 (7,145,710-12,450,758)x1	5305 kb	Unknown	ASD, VSD with PS. Discrete facial dysmorphism, a shield shaped chest with supranumerary nipples. Hyperactivity, impulsiveness with moderate ID.
18	4 y	F	arr [hg19] 7q34q36.2 (141,383,311-154,467,488)x1	13 Mb	*de novo*	DD, speech impairment and Facial dysmorphism.
20	6 y	M	arr [hg19] 2q33.1q33.3 (198,383,221-206,943,477)x1	8560 kb	*de novo*	Severe ID, facial dysmorphism with absence of speech and autistic spectrum behavior.
34	25 months	F	arr [hg19] 7q11.23 (72,726,572-74,133,332)x3	1406 kb	Maternally inherited	Cor pulmonare associated with a DD and speech delay,facial dysmorphism, genu valgum.
36	14 y	F	arr [hg19] 8q24.3 (143,631,709-146,274,835)x3,16p13.3(96,766-1,850,720)x1	2643 kb and 1754 kb	Unknown	Severe ID, facial dysmorphism clubfoot, short stature and behavior problems characterized by self-mutilation.
37	6 y	M	arr [hg19] 22q11.21 (18,706,001-21,464,119)x1	2758 kb	*de novo*	Speech delay, severe ID, VSD, DD, Facial dysmorphism, ear abnormalities.
39	4 y	F	arr [hg19] 10p15.3p14 (136,361-11,073,839)x1	10 Mb	*de novo*	DD, neonatal hypotonia, and absence of speech development. Facial dysmorphism and clinodactyly.
45	2 y	M	arr [hg19] 17q21.31q21.32(44,156,499-45,152,416)x1	995 kb	*de novo*	DD, epilepsy, facial dysmorphism consisting of hypertelorism, low set ears, hypotonia and sparse hair.

### Clinical description of our cohort

**Figure 1:**
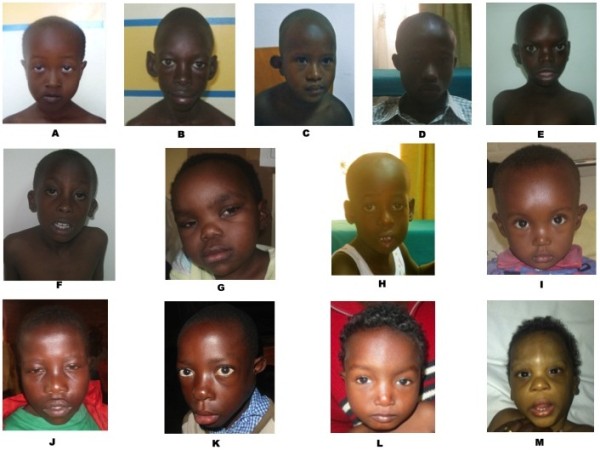
**Pictures of the 13 patients with pathogenic CNVs. A**. Patient 1 (trisomy 18p); **B** Patient 6 (william- Beuren syndrome); **C** Patient 13 (del 6q16.1q21); **D** Patient 14 (del 22q11.2); **E** patient 16 (dup 1p35.3 p31.3); **F** patient 17 (del 8p23.1); **G** patient 18 (del 7q34q36.2); **H** patient 20 (del 2q33.1q33.3); **I** patient 34 (dup 7q11.23); **J** patient 36 (dup 8q24.3/del 16p13.3); **K** patient 37 (del 22q11.21); **L** patient 39 (del 10p15.3p14); **M** patient 45 (del 17q21.3q21).

Among fifty patients, 14 (28%) had a global development delay whereas 36 (72%) patients presented intellectual (ID) disability. Patients with ID were of different degrees: mild (6 patients), moderate (11 patients), severe (16 patients) and 3 with profound ID. All patients presented MCA. The most common clinical features were craniofacial dysmorphism found in 41 patients, limbs abnormalities in 14 patients, congenital heart defect in 15 patients, microcephaly in 9 patients and external genitalia abnormalities in 4 patients (Table 2).In this report, we described case by case each patient with clinical relevant copy number polymorphism (Figure 1).

**Table 2:** Clinical characteristics of the 50 Rwandans patients with ID/DD and MC

**Characteristics**	**Number (percentage)**
Gender	
Male	33 (66%)
Female	17 (34%)
Age groups	
< 5years	24 (48%)
5-15 years	19 (38%)
> 15 years	7 (14%)
Intellectual disability	
Present	34 (68%)
Mild	4 (8%)
Moderate	13 (26%)
Severe	16 (32%)
Profound	1 (2%)
Not evaluated	16 (32%)
Development delay	
Absent	6 (12%)
Present	44 (88%)
Facial dysmorphism	
Absent	9 (18%)
Present	41 (82%)
Congenital heart defect	
Absent	35 (70%)
Present	15 (30%)
Hand and limb abnormalities	
Absent	36 (72%)
Present	14 (28%)
Uro-genital malformation	
Absent	46 (92%)
Present	4 (8%)
Epilepsy	
Absent	43 (86%)
Present	7 (14%)
Microcephaly	
Absent	41 (82%)
Present	9 (18 %)

Patient 1 was a 6-year-old girl patient who consulted for moderate ID associated with an atriventricular septal defect (AVSD) and atrio septal defect (ASD). Clinical evaluation showed minor facial dysmorphic features characterized by hypertelorism, low set ears, downslanted palpebral fissures, flat midface, a small mouth, bilateral incomplete transverse simian crease and clinodactyly. Her karyotype revealed an extrachromosomal marker. The karyotypes of her parents were normal. The array-CGH showed a duplication of 14 Mb in the 18p11.32p11.21 region and a duplication of 2925 kb in 18p11.21-q11.2 regions. However, the 1,1 Mb pericentromeric region of 18p (14,241,744–15,345,079) showed normal values.

Patient 6 was an 11-year-old boy with moderate ID. He had a large face, large lobule, periorbital fullness, wide mouth with full lip and a small jaw. He presented a friendly behavior with old-looking appearance according to his age. Echocardiography showed a mitral valve prolapse. The array-CGH revealed a deletion of 1442 kb localized in the Williams-Beuren syndromic region (7q11.23).

Patient 13 presented a deletion 6q16.1q21 of 14 Mb size. He was a boy aged 6 years who consulted for absence of speech with severe ID. Clinical features included ear abnormalities, microcephaly (head circumference of 47 cm equals to -4 SD), hypertelorism, short philtrum, cupped ears, brachydactyly, wide spaced nipple, bilateral cryptorchidism and autistic-like behavior. He had a good appetite but was underweighted (15 kg equal to – 2SD).

Array-CGH revealed a 22q11.22 deletion of 2758 kb in patient 14 and patient 37. Patient 14 (15 years) presented mild ID, hypotonia at birth, dysmorphic features characterized by hypertelorism, narrow palpebral fissures, wide nasal bridge, small ears with overfolded helix, short philtrum and a large upper lip. He had hypernasal speech, a short stature without any congenital cardiac defect. The second patient (patient 37) a 6-year-old boy consulted for speech delay, severe ID and ventricular septal defect (VSD). He had DD, dysmorphic features marked by hypertelorism, attached ear lobe, preauricular pits, cupped ears and bulbous nasal tip.

Patient 16 had a large interstitial duplication 1p 35.3p31.3 of 34,354 Mb. The 10-year-old boy had moderate ID with a history of DD, anxiety and hearing impairment. He presented facial dysmorphism characterized by hypertelorism, strabismus, depressed nasal bridge, arched eyebrows, midface hypoplasia, anteverted nostrils macroglossia, teeth malposition and clinodactyly. We noticed unilateral blepharospam on the right side.

Patient 17 was a 9-year-old boy in whom array-CGH revealed a deletion in the 8p23.1 region. His clinical features consisted of a congenital cardiac defects characterized by atrial septal defect (ASD), ventricular septal defect (VSD) with a pulmonary stenosis (PS). He had discrete dysmorphic features characterized by overfolded ears, a shield shaped chest with supernumerary nipples. His behavior disorders consisted of hyperactivity and impulsiveness with moderate ID.

Patient 18, a 4-year-old girl consulted our department for DD, speech impairment and facial dysmorphic features characterized by coarse face, hypertelorism, epicanthic folds, mild synophris, deep set eyes, narrow palpebral fissures, bulbous nasal tip, low set and misshapen ears. The a-CGH detected a 7q34q36.2 deletion with a large deletion of 13 Mb.

Patient 20 presented a 2q33.1-q33.3 deletion. The patient was 6-year-old boy who had severe ID, facial dysmorphism consisting of a large face, dental abnormalities and high arched palate with absence of speech and autistic spectrum behavior.

Patient 34, a 25-month-old girl had maternally inherited 7q11.23 duplication She was referred for management of a cor pulmonale associated with a DD and speech delay, dysmorphic features dominated by hypertelorism, prominent forehead, low set and small ears. She also had genu valgum. The mother had a past medical history of delayed speech, moderate intellectual disability. Her phenotype consisted of low set and small ears with prominent forehead.

Patient 36 showed unusual chromosomal abnormalities with the co-occurrence of a 8q24.3 duplication and a 16p13.3 deletion. The patient a 14 year old girl, was referred for severe ID, facial dysmorphism with hypertelorism, high forehead, short philtrum, broad nasal bridge, low set ears, clubfoot, short stature and behavior problems characterized by self-mutilation.

A large 10p15.3p14 deletion was found in a 4-year-old girl (patient 39). She presented DD, neonatal hypotonia, and absence of speech development. Facial dysmorphism consisted of hypertelorism, high arched eyebrows, short philtrum, thin upper lips, low set ears, and clinodactyly.

Patient 45 had a microdeletion 17q21.31 of 995 kb. He was a 2-year-old boy with DD, epilepsy, facial dysmorphism consisting of hypertelorism, low set ears, hypotonia and sparse hair.

## Discussion

Our study is the first largest cohort of East–African patients with DD/ID characterized by array-CGH. In our cohort of 50 Rwandan patients we detected 14 genomic imbalances yielding a diagnosis rate of 26%, which is higher than other reported array-CGH studies in patients with DD/ID [12-14]. However, the high detection rate might be explained by the selection bias of our patients characterized by more discriminatory criteria than in reported previous studies. However, our result are comparable to those reported by Iourov and al [15], where they concluded that an application of array-CGH to highly selected patients is able to reveal an impressive detection rate of structural genome variations.

**Figure 2:**
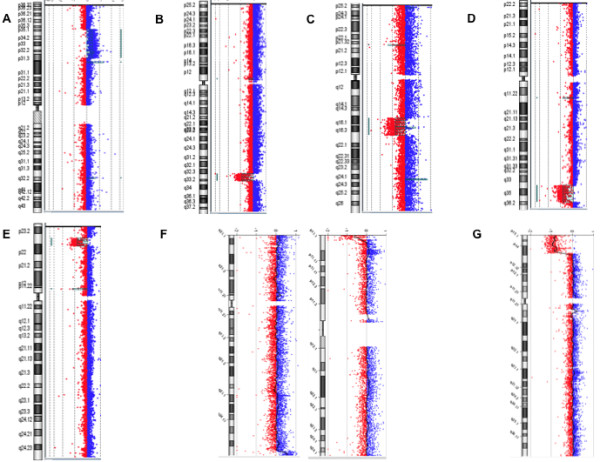
**Results of array-CGH analysis. A** large duplication 1p35.3p31.3 found in patient 16; **B** chromosome 2q33.1q33.3 deletion found in patient 20. **C** deletion 6q16.1q21 (patient 13). **D** deletion 7q34q36.2 found in patient 18. **E** result of patient 17 with a deletion 8p23.1. **F** duplication 8q24.3 associated with a deletion 16p13.3 found in patient 36. **G** large deletion 10p15.3p14 found in patient 39.

We reported only clinical relevant CNVs (Figure 2) in our patients in whom 6 had known syndromes, and 8 had rare but previously reported CNVs. Interestingly, one patient was the second reported in the literature presenting concomitant 8q24.3 duplication and 16p13.3 deletion [16], and the first patient with this abnormality to be characterized by array-CGH.

### Known syndromes

Five of our patients presented known syndromes associated with distinct phenotype. Among them, two presented rearrangement in the Williams-Beuren critical region, patient 6 with a de novo 7q11.23 deletion of 1.442 MB and patient 30 with a maternally inherited duplication 7q11.23 of 1.4 MB. The deletion encompassed 30 genes while the duplication involved 28 genes. CNV encompassed the candidate gene ELN, LIMK1, GTF2I, and GTF2IRD1 mainly involved in clinical features found in this syndrome. Deletion of ELN gene is clearly involved in the vascular anomalies and other connective tissue abnormalities in William-Beuren syndrome (WBS) [17]. LIMK1, GTF2I and GTF2IRD1 genes have been related to aspects of cognitive delay [18,19]. Duplication of the 7q11.23 has been reported to be associated with severe delay in expressive language suggesting that specific genes within this region can influence language [20]. This suggestion is consistent with clinical features of our patient as at 2 years of age she had not yet developed speech and even her mother had a history of speech delay.

In our study, we found two patients (patients 11 and 33) with deletions in the 22q11.22 region. Deletion 22q11.22 is characterized by its broad spectrum phenotype [21]. In our study one patient had a velopharyngeal insufficiency with moderate intellectual disability while the other patient presented a VSD and severe ID. Both patients had ears abnormalities and this sign has been suggested as the most prevalent in African-American patients with 22q11.22 deletion [22].

In addition, array-CGH revealed a deletion of 5 Mb in the 8p23.1 region in patient 17. Clinical features were consisting with the 8p23.1 deletion syndrome. The deletion encompassed the GATA4 gene, which encodes for zinc finger transcription factor and is considered as a likely candidate for the cardiac malformation [23,24]. However, this is consistent with our patient since he is born with a complex congenital heart defect including ASD.

Patient 45 presented a deletion in the 17q21.31q21.32 region of about 1 Mb, that encompassed about 30 genes including KANSL1. The 17q21.31 deletion syndrome has been suggested as a single gene disorder caused by haploinsufficiency of KANSL1 gene [25]. Most case of 17q21.31 deletions reported map to large clusters of flanking low copy repeats (LCRs), suggesting that the deletions are stimulated by non-allelic homologous recombination (NAHR) [26].

The 17q21.31 genomic interval contains a common 900 kb inversion polymorphism, resulting in a haplotype block with two highly divergent haplotypes designated H1 and H2. The H2 haplotype is enriched in Europeans, and carriers are predisposed to the 17q21.31 microdeletion syndrome as a result of NAHR between directly oriented segmental duplications mapping on the inverted chromosome. An ancestral H2 haplotype (H2**′**) lacking these duplications was identified and Steinberg and al suggested that it arose in Eastern or Central Africa [27]. Our patient is the second known African American reported with 17q21.31 microdeletion [28]. Morever, the breakpoints of our patient’s deletion do not map inside the recurrent minimal 424 kb critical region deleted found in patients reported by Koolen and al [26].

### Rare reported CNVs

In our cohort, nine patients presented previously reported but rare CNVs. Patient 1 had a trisomy for the short arm of chromosome 18 originating from a small supernumerary marker chromosome (sSMC). Interestingly, Trisomy 18p caused by sSMC has been previously reported in six patients [29]. The phenotype of our patient is in agreement with previously described patients with such similar chromosome abnormality [30].

An interstitial 6q 16.1q21 deletion of about 14 MB encompassing nearly 98 genes was detected in patient 13. Suggested candidate genes involved in the central nervous system (CNS) development were EPHA7 and GRIK2. EPHA7 encodes for a member of the ephrin family implicated in mediating developmental events, particularly in the nervous system [31]. GRIK2 encodes for a glutamate receptor that has been associated with autistic-spectrum-disorders and neuropsychiatric diseases [32]. The CNV encompassed genes involved in regulation of feeding behavior such as SIM1, MCHR2 and POU3F2. SIM1 haploinsufficiency has been proposed to cause Prader-Willi-like phenotype in 6q deletions [33-35]. Moreover, some features of the Prader-Willi syndrome such as obesity, are missing in our patient.

Patient 13 had a large duplication in the region 1p35.3p31.3. Chromosome 1p duplication is a very rare rearrangement with a variety of clinical features and is associated with short-term survival. To date, only 20 patients have been described in the literature [36]. Due to the high number of genes, approximately 600 genes are included in the duplication region, it was difficult to correlate the genotype to the phenotype. However, patients with mutations in genes mapping in the 1p35.3p31.3 region presented similar clinical features with those of our patient. Patients with homozygous mutations in SNIP1 or COL9A2 had some cranio-facial dysmorphic features observed in our patient. Normally, patients with SNIP1 mutations present bulbous nose, wide mouth and macroglossia. Moreover, in our patient, the psychomotor delay is less severe than those patients [37]. Mutations in COL9A2 gene cause the autosomal recessive Stickler syndrome type 5. Patients affected by this syndrome present some of our patient’s clinical features such as midface hypoplasia, anteverted nostrils and hearing impairment [38]. In addition, heterozygous mutations in the GJB3 and KCNQ have been associated with autosomal dominant hearing loss [39,40]. Genes linked to ID mapped in the duplicated region are GLUT1, ST3GAL3. Heterozygous mutations in the GLUT1/SLC2A1 gene, occurring de novo or inherited as an autosomal dominant trait, result in cerebral energy failure and a clinical condition termed GLUT1-deficiency syndrome (GLUT1-DS). Clinical features usually comprise motor developmental delay and intellectual disability, seizures with infantile onset, deceleration of head growth often resulting in acquired microcephaly, and a movement disorder with ataxia, dystonia, and spasticity [41]; whereas mutations in the ST3GAL3 cause nonsyndromic autosomal recessive ID [42]. Recently, Crane J. and collaborators [43], using linkage analysis study, suggested DLGAP3 as a candidate gene for Tourette syndrome. Except for the presence of tics in our patient, other signs of this syndrome were missing.

Patient 18 showed a large interstitial deletion of 12.2 MB in the 7q34q36.2 region. This patient showed similar dysmorphic features as other patients carrying this deletion such as facial coarse face and bulbous nose [44-46]. Candidate genes included in the deletion are CNTNAP2, KCNH2 and NOBOX2. CNVs in the CNTNAP2 gene have been correlated with moderate and profound ID, speech impairment, seizures and dysmorphic features [47]. Even though our patient had a normal cardiac and brain evaluation, the deletion encompass the KCNH2 gene encoding the **α**-subunit of the hERG-1 voltage-gated K + channel expressed in heart and brain tissues [48] and this gene was suggested as responsible of the Long QT syndrome in patients with 7q34 deletion [45]. The NOBOX2 associated with premature ovarian failure in three patients with 7q334-36 deletion is also deleted [49] , but our patient is too young to assess this sign.

Patient 20 had a de novo deletion 2q33.1-q33.3 of 8.5 Mb which encompassed about 118 genes. The deletion included the SATB2 gene, coding for a DNA-binding protein that regulates gene expression by influencing chromatin organization and structure and orchestrating the transcription of several genes [50]. Haploinsufficiency of SATB2 is responsible for several of the clinical features such as craniofacial patterning, severe developmental delay and tooth abnormalities associated with 2q32q33 microdeletion syndrome [51,52].

Patient 39 had a large deletion of 10 Mb in the 10p15.3p14 region. The deletion encompassed 86 genes. Genes expressed in the brain are ZMYND11, DIPC2, ADARB2, and GATA3. ZMYND11 and DIPC2 genes are the most commonly deleted in a cohort of 19 patients with 10p15 deletion previously described by DeScipio et al. [53]. They are expressed in various tissues including the brain. However, little is known about their function, making direct genotype/phenotype correlation currently unclear. ADARB2 a member of the double-stranded RNA adenosine desaminase family of RNA-editing enzymes is expressed only in selected region of the brain such as amygdala and thalamus and may probably play a regulatory role in tRNA editing in mammalian brains [54]. The GATA3 gene haploinsufficiency is the cause of hypoparathyroidism with sensorineural deafness and renal dysplasia also known as Barakat syndrome [55]. Our patient had no sign of hypoparathyroidism or renal dysplasia but presented sensorineural deafness.

### Combined abnormalities

Patient 36 showed unusual abnormalities with the co-occurrence of 8q24.3 duplication and 16p13.3 deletion. These associated abnormalities were first reported to result from an unbalanced translocation [16]. However, our patient is the first to be characterized by a-CGH. The 2 Mb duplication in the 8q24.3 region encompassed around 156 genes. The partial trisomy 8q has been described to be associated with autism disorders [56]. The deletion of 1.754 Mb in the 16p13.3 deletion is located in the ATR-16 syndromic region and includes 136 genes. ATR-16 is defined as a contiguous gene syndrome resulting from hemizygous loss of the α-globin gene cluster and genes involved in ID [57]. Haploinsufficiency of SOX8 a transcriptional regulator strongly expressed in brain, is thought to be responsible for the ID of ATR-16 syndrome [58]. The hematological evaluation of our patient was not possible but she presented severe ID and a history of DD. She also presented clinical features found in other patients affected by this syndrome such as hypertelorism, high forehead, broad nasal bridge and clubfoot [59].

## Conclusion

This research highlights the contribution of genetic factors in the etiology of DD/IDD and MCA, especially the implication of chromosomal abnormalities with an array-CGH detection high rate of 28%. This study showed the importance to implement genetics services in low-middle income countries; as array-CGH is becoming cheaper, it can also be considered as the first-line analysis in DD/ID and MCA in those countries, because it has the advantages of higher diagnosis rate than the conventional karyotype.

To the best of our knowledge, the present study is the first one done in East-African patients with DD/IDD and MCA.

## Abbreviations

ID: Intellectual disability; IQ: Intelligence quotient; DD: Development delay; MCA: Multiple congenital abnormalities; Array-CGH: Array comparative genomic hybridization; RNEC: Rwandan National Ethics Committee; MLPA: Multiplex ligation-dependent probe amplification; FISH: Fluorescence in situ hybridization; CNV: Copy number variation; AVSD: Atriventricular septal defect; ASD: Atrial septal defect; VSD: Ventricular septal defect; PS: Pulmonary stenosis; WBS: William Beuren syndrome; sSMC: A small supernumerary marker chromosome; WBI: Wallonie Bruxelles International; CUD: Coopération Universitaire au Développement.

## Competing interests

The authors declare that they have no competing interests.

## Authors' contribution

AU designed the study project. AU and JH were involved in data collection. JHC, ACH, MJ and VD supervised laboratory techniques and results interpretation. EKR performed cardiac ultrasound in all patients. AU drafted the manuscript. LM and VB supervised and coordinated all project activities. All authors read and approved the manuscript.

## Pre-publication history

The pre-publication history for this paper can be accessed here:

http://www.biomedcentral.com/1471-2350/15/79/prepub

## Supplementary Material

Additional file 1: Figure S1 MLPA and FISH results. Click here for file
