# Supplementary material for: Array-CGH analysis in Rwandan patients presenting development delay/intellectual disability with multiple congenital anomalies
Source: BMC Med Genet. 2014 Jul 12;15:79. doi: 10.1186/1471-2350-15-79 (PMC4123504; doi:10.1186/1471-2350-15-79)
Supplement: Additional file 1: Figure S1 — MLPA and FISH results. [file 1471-2350-15-79-S1.pdf]

MLPA results

Results:

**P036-E1**	**AB13GS307_(130121-0046_P036-E1_F_P)_B04_ABI**			**06/12/2013**	**All vs all**	
	duplication	08	q	ZC3H3 (KIAA0		361
	deletion	16	p	POLR3K		242
**P070-B1**	**AB13GS307_(130121-0046_P070-B1_F_P)_G04_AB**			**06/12/2013**	**All vs all**	
	duplication	08	q	RECQL4		179
	deletion	16	p	DECR2		427

Analysis Mode: All vs all

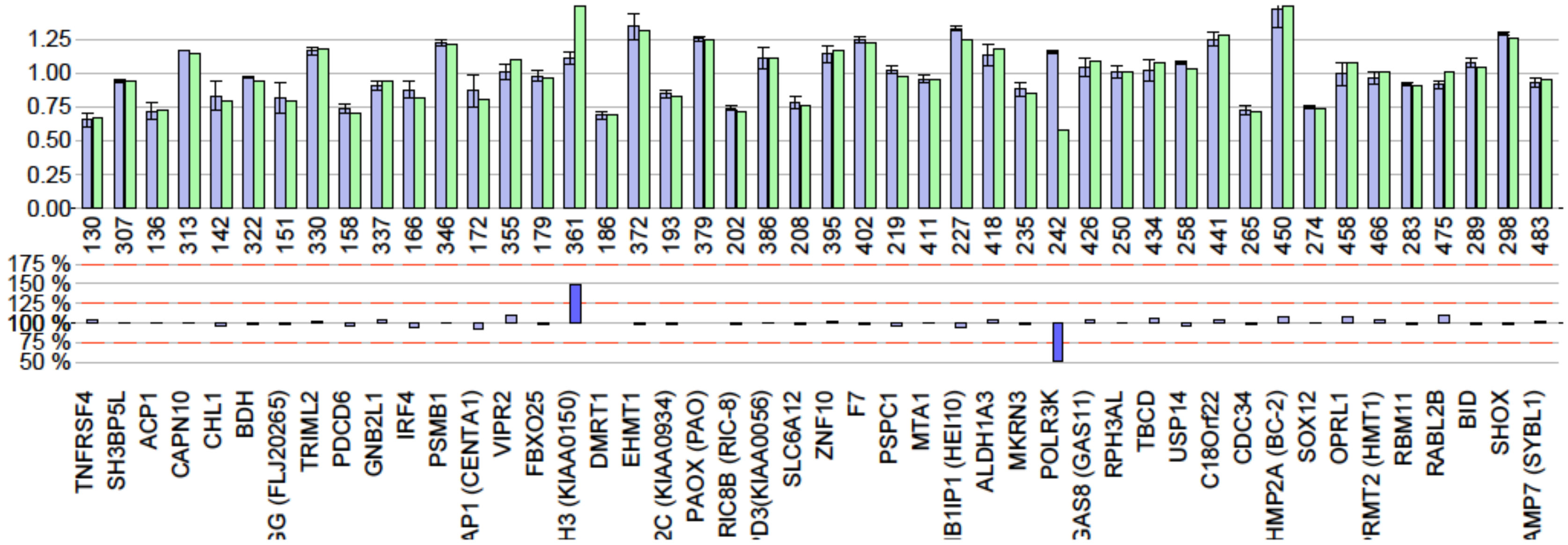

Deletion 8q24.3/16p13.3

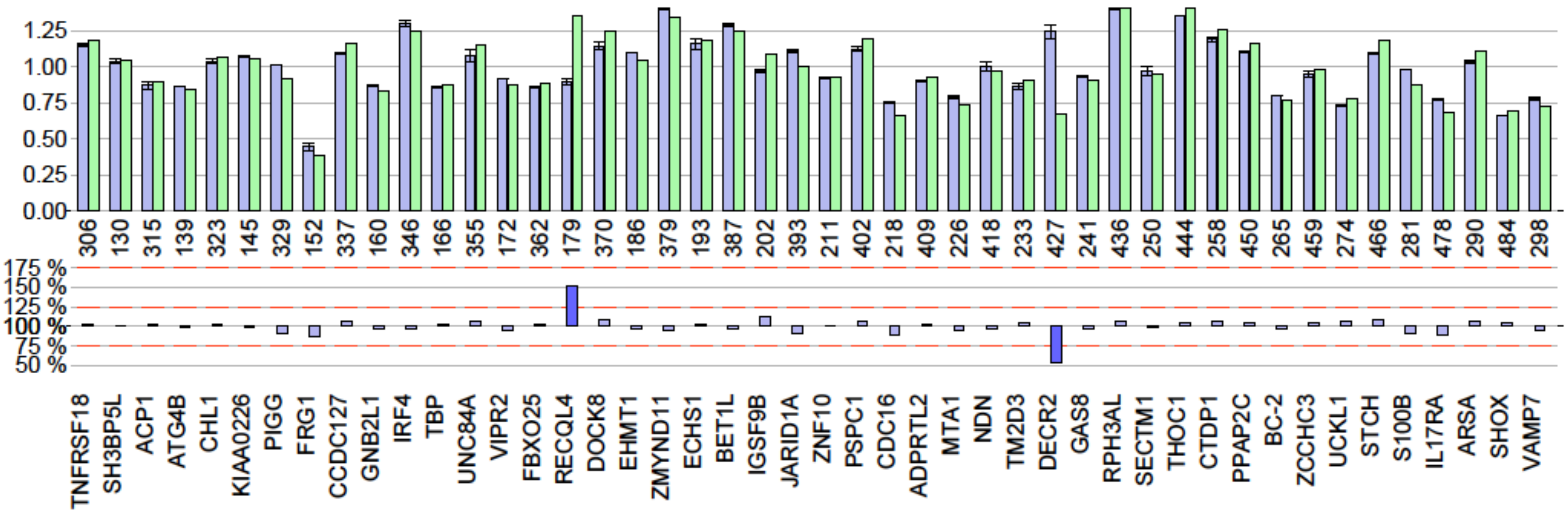

Deletion 8q24.3/16p13.3

Deletion 22q11.21

Results:

**P245-A2**	**AB13GS307_(130121-0049_P245-A2_M_P)_C03_AB**	**06/12/2013**			**All vs all**	
	deletion	22	q11.21	CLDN5		196
	deletion	22	q11.21	GP1BB		208
	deletion	22	q11.21	SNAP29		373

**Resultfile:**	AB13GS307_(130121-0049_P245-A2_	P245-A2	06/12/2013 11:54:00	**total peak area:**	258917
**Control:**	AB13GS307_(ctrl4-12062013_P245-A2	P245-A2	06/12/2013 11:54:00	compl. TV	UA [06/12/2013 14:08:20]
**Control:**	AB13GS307_(ctrl3-12062013_P245-A2	P245-A2	06/12/2013 11:22:00	compl. TV	UA [06/12/2013 14:07:24]

data correction: on
DNA dosage: ok
total peak area: ok
max peak area: ok
max peak height: ok

Analysis Mode: All vs all

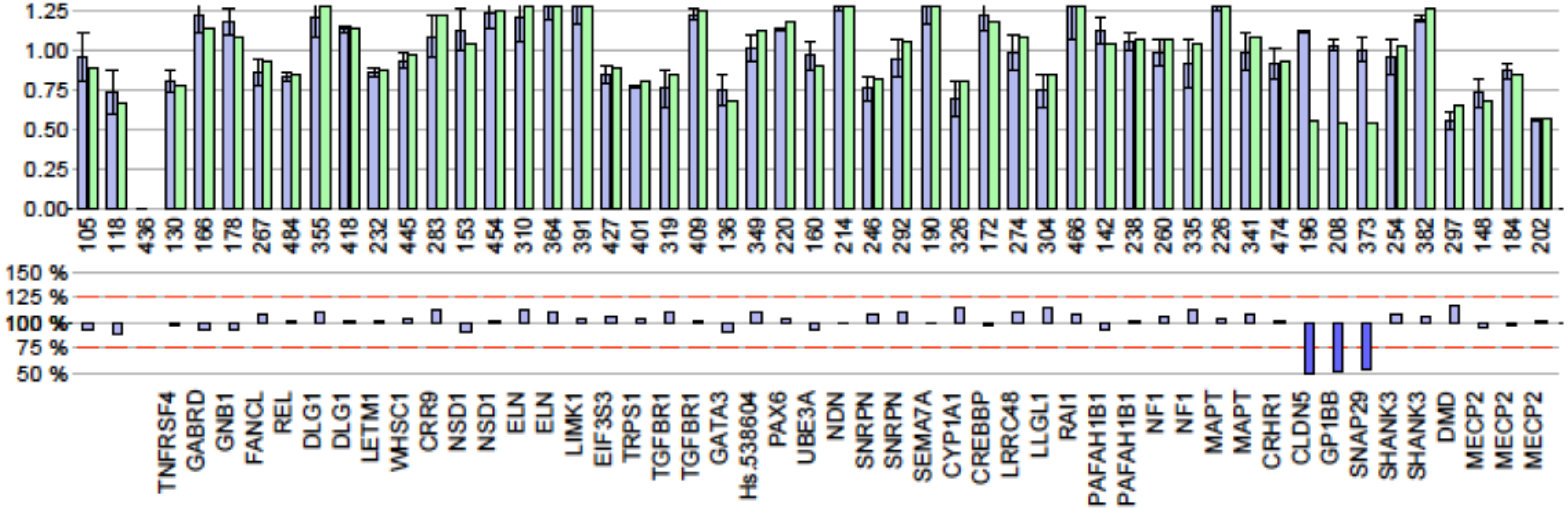

Results:

**P036-E1**	**AB13GS307_(130121-0066_P036-E1_F_P)_A04_ABI**			**06/12/2013**	**All vs all**	
	deletion	10	p	DIP2C (KIAA0!		193
**P070-B1**	**AB13GS307_(130121-0066_P070-B1_F_P)_F04_ABI**			**06/12/2013**	**All vs all**	
	deletion	10	p	ZMYND11		379
**P245-A2**	**AB13GS307_(130121-0066_P245-A2_F_P)_B03_ABI**			**06/12/2013**	**All vs all**	
	deletion	10	p15.1	GATA3		136
	deletion	10	p15.1	Hs.538604		349

**Resultfile:**	AB13GS307_(130121-0066_P036-E1_	P036-E1	06/12/2013 11:54:00	**total peak area:**	288301
**Control:**	AB13GS307_(ctrl1-12062013_P036-E'	P036-E1	06/12/2013 11:54:00	compl. TV	UA [06/12/2013 13:41:17]
**Control:**	AB13GS307_(ctrl2-12062013_P036-E'	P036-E1	06/12/2013 11:54:00	compl. TV	UA [06/12/2013 13:43:11]

**data correction:**	on
**DNA dosage:**	ok
**total peak area:**	ok
**max peak area:**	ok
**max peak height:**	ok

Deletion 10p15.3p14

Analysis Mode: All vs all

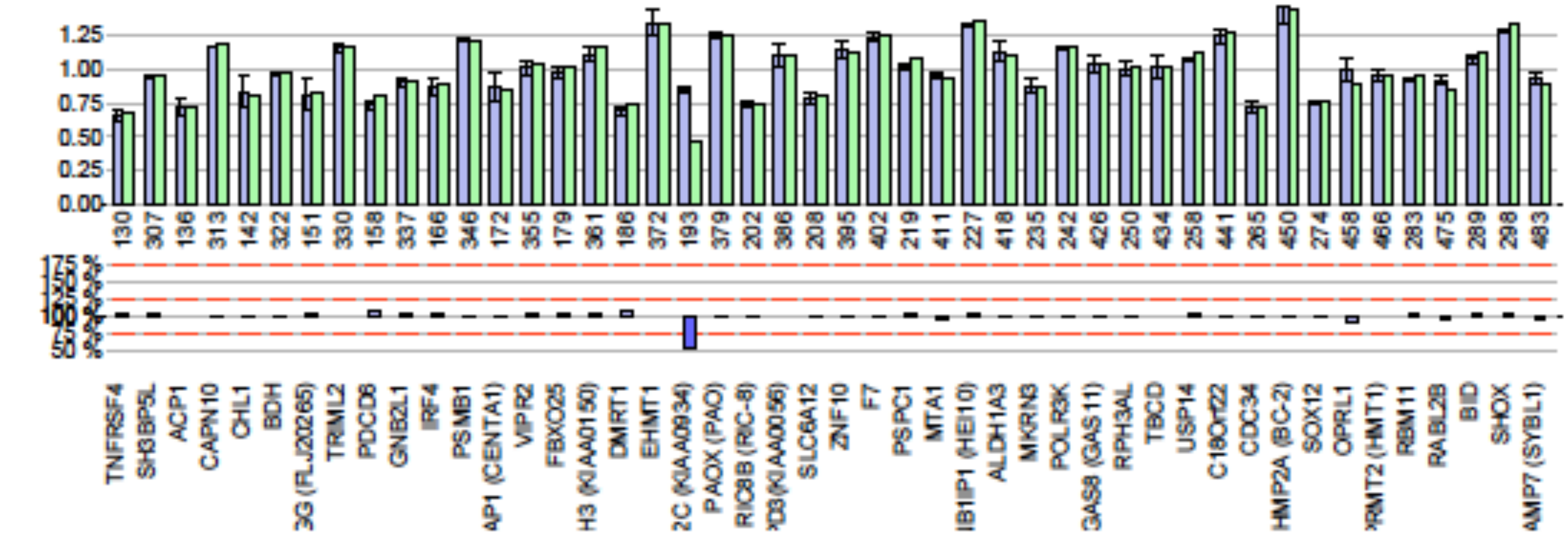

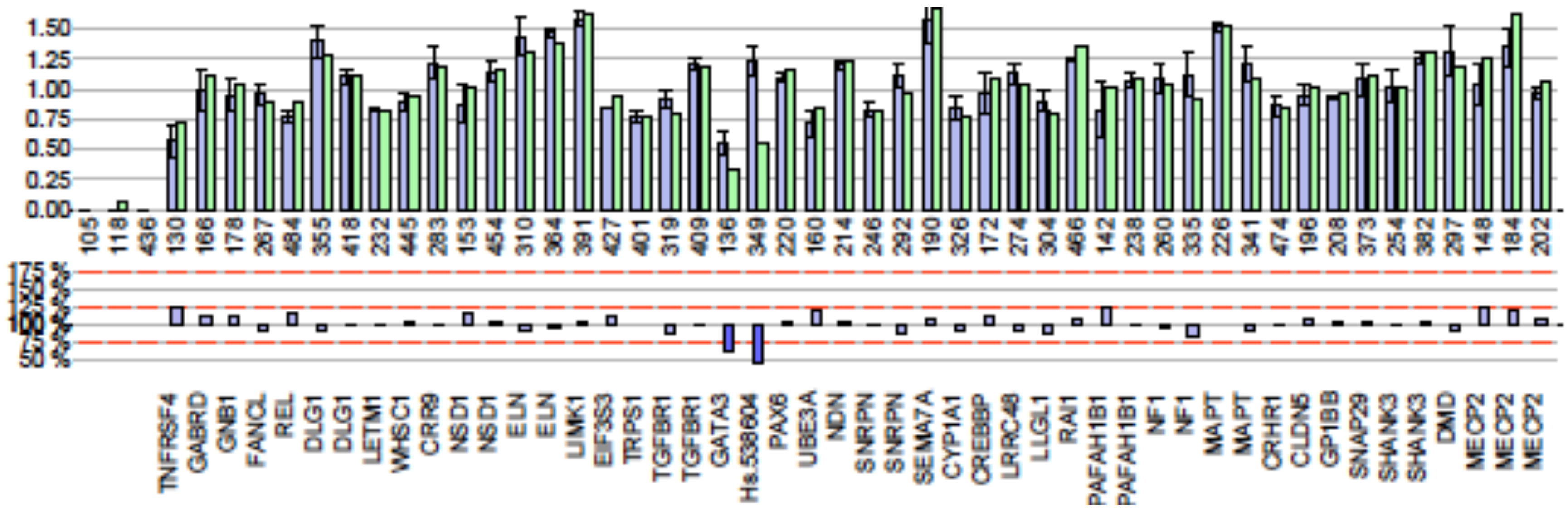

Deletion 10p15.3p14

P245-A2 AB13GS236_(130121-0103_P245-A2_F_P)_H10_AB 05/03/2013 All vs all

duplication 07 q11.23 LIMK1 391

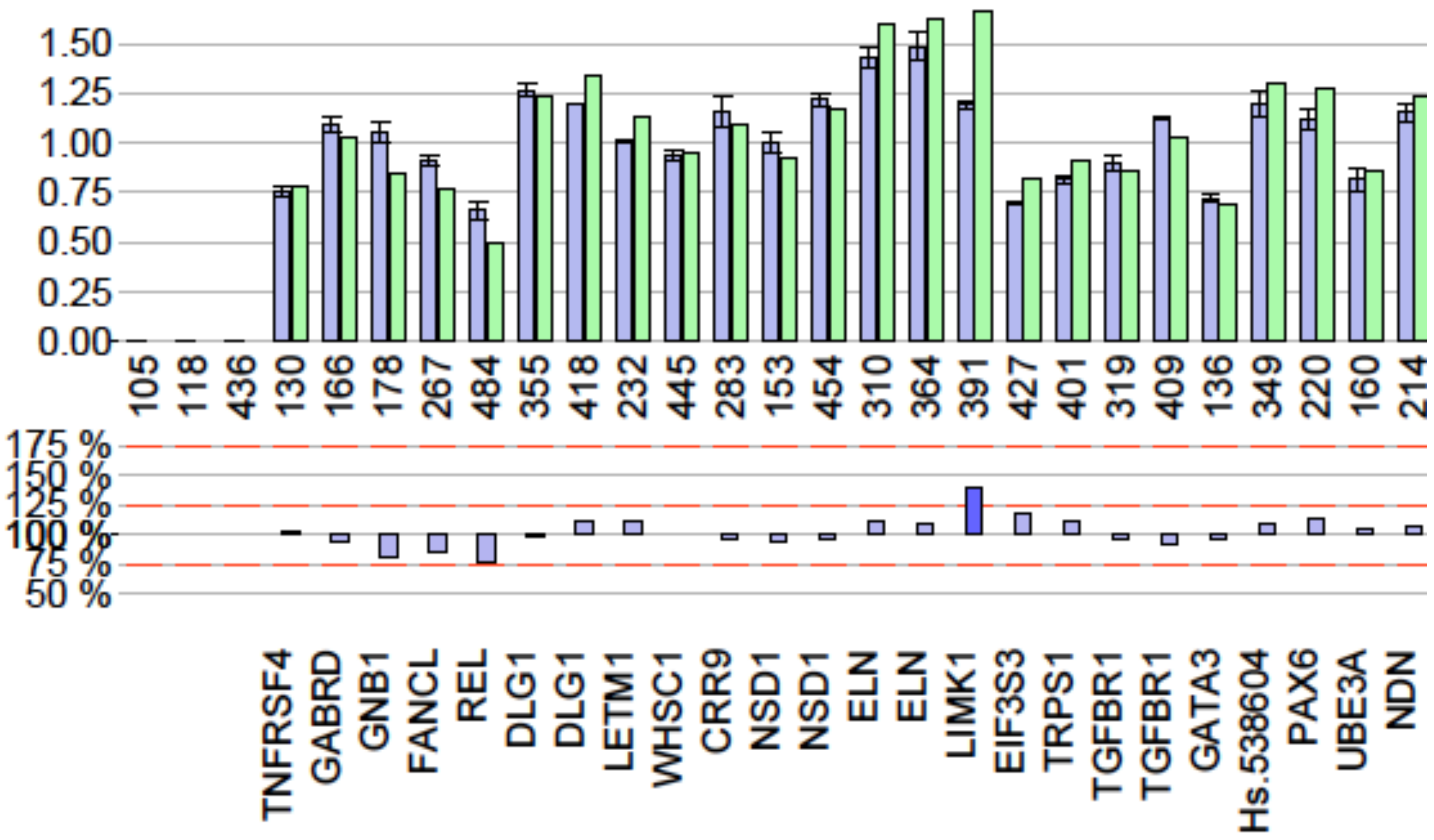

Duplication 7q11.23

Results:

**P245-A2**	**(110408-0068P_P245-A2_M_P)_H04_AB11GS265_2**	**06/29/2011**	**All vs all**		
	deletion	07	q11.23	ELN	310
	deletion	07	q11.23	ELN	364
	deletion	07	q11.23	LIMK1	391

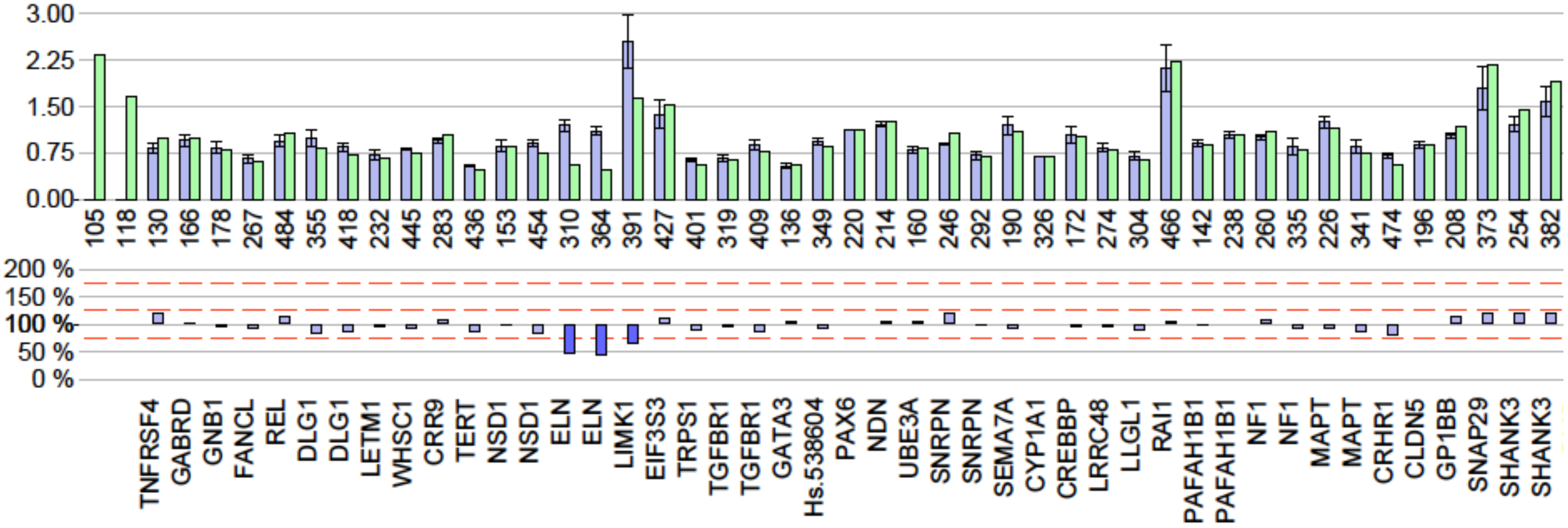

Deletion 7q11.23

P070-B1 (121131-0110P_P070-B1_F_P)_H09_AB12GS118_2(03/14/2012 All vs all

duplication 18 p THOC1 444

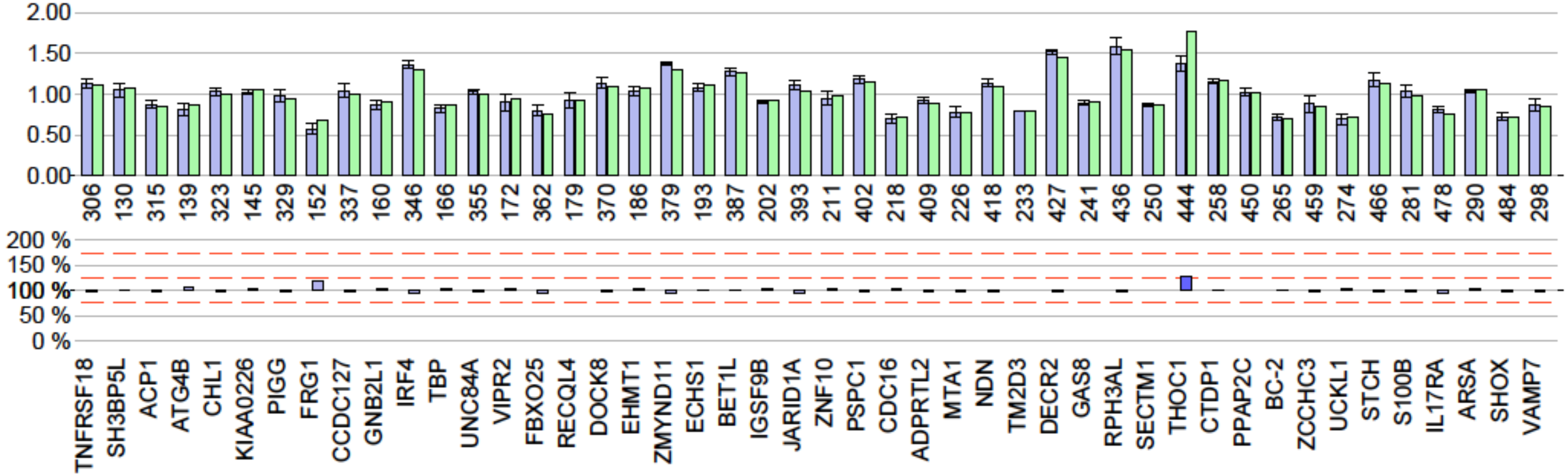

Trisomy 18p

FISH Results

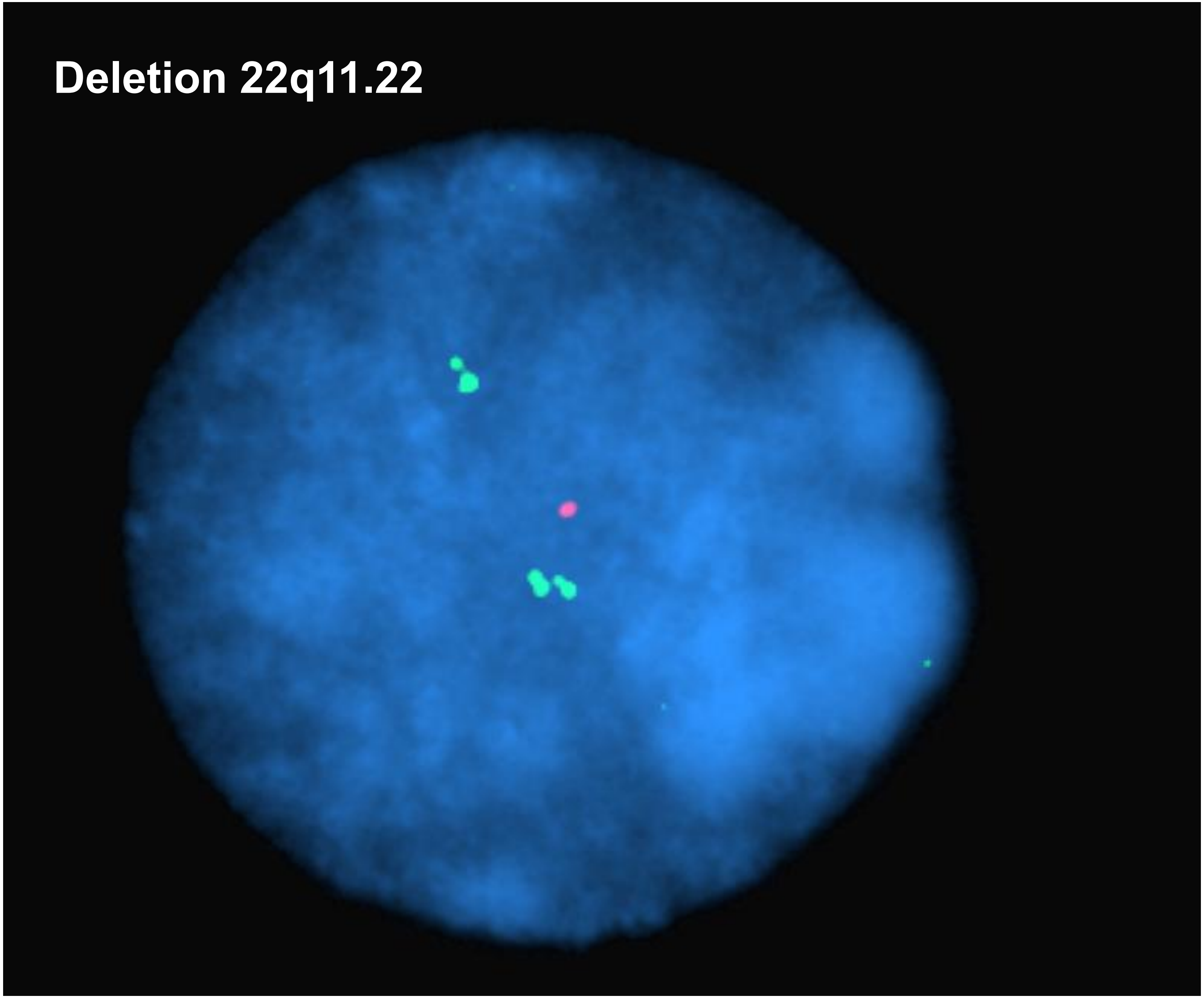
Deletion 22q11.22

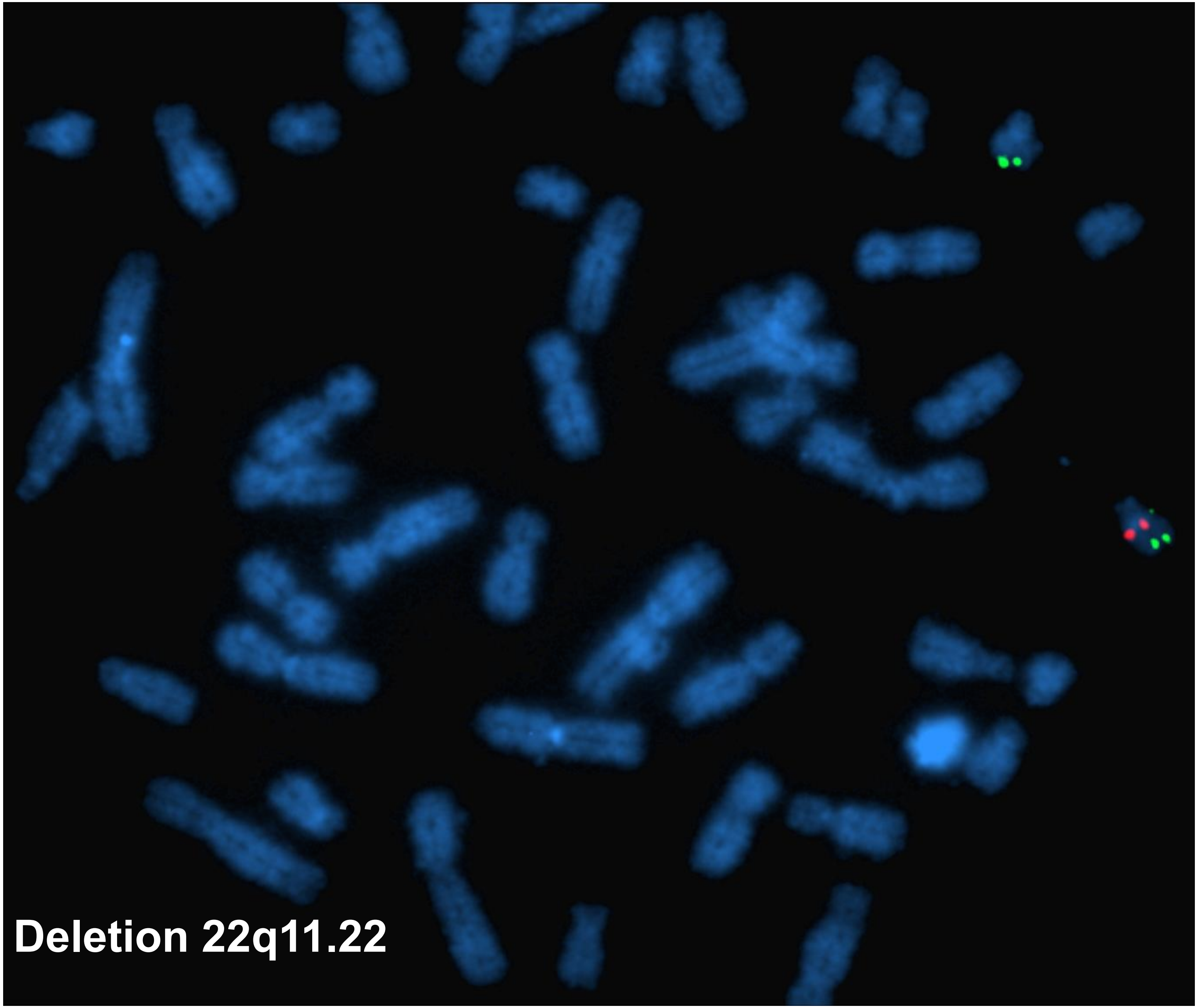
Deletion 22q11.22

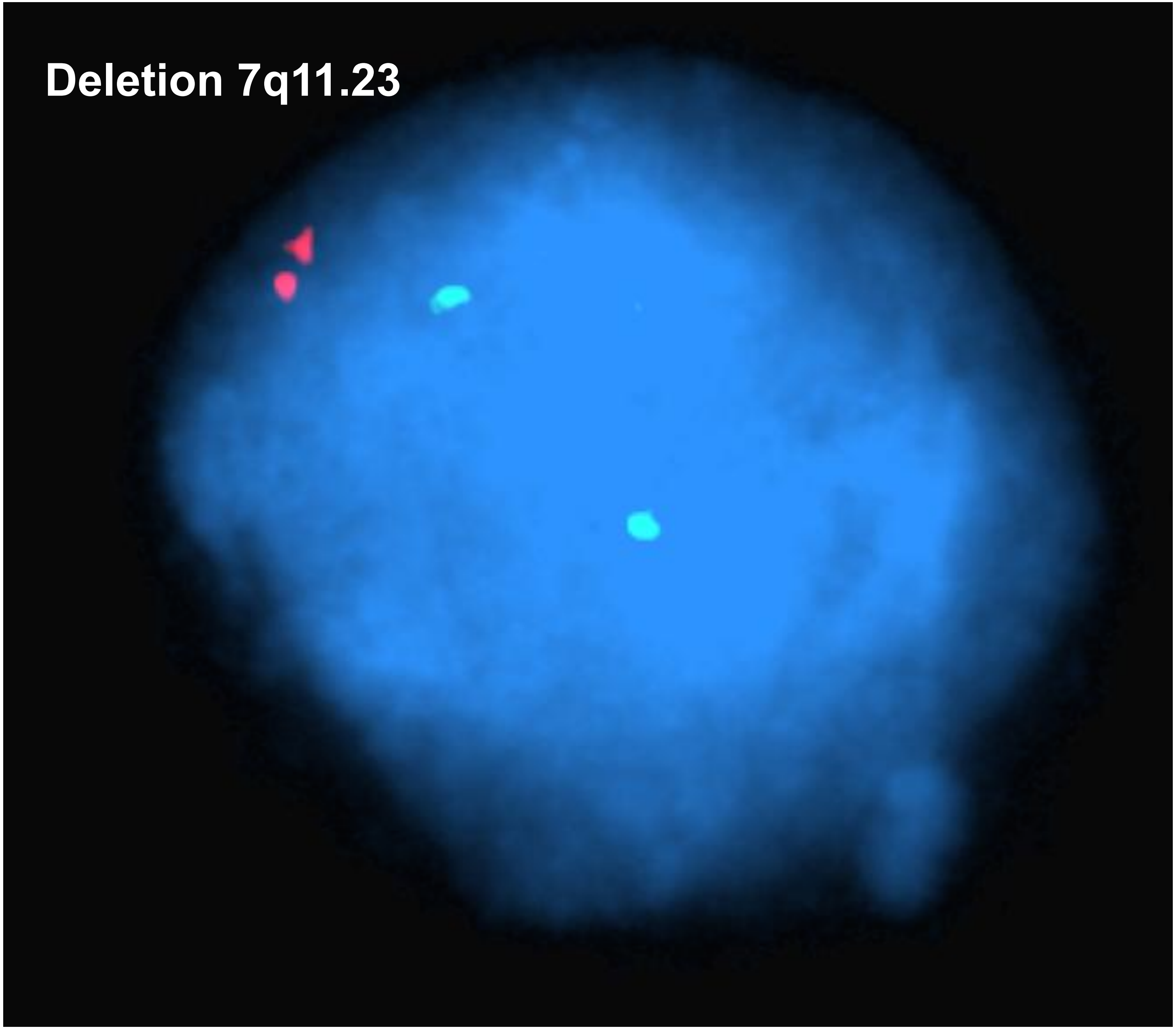
Deletion 7q11.23

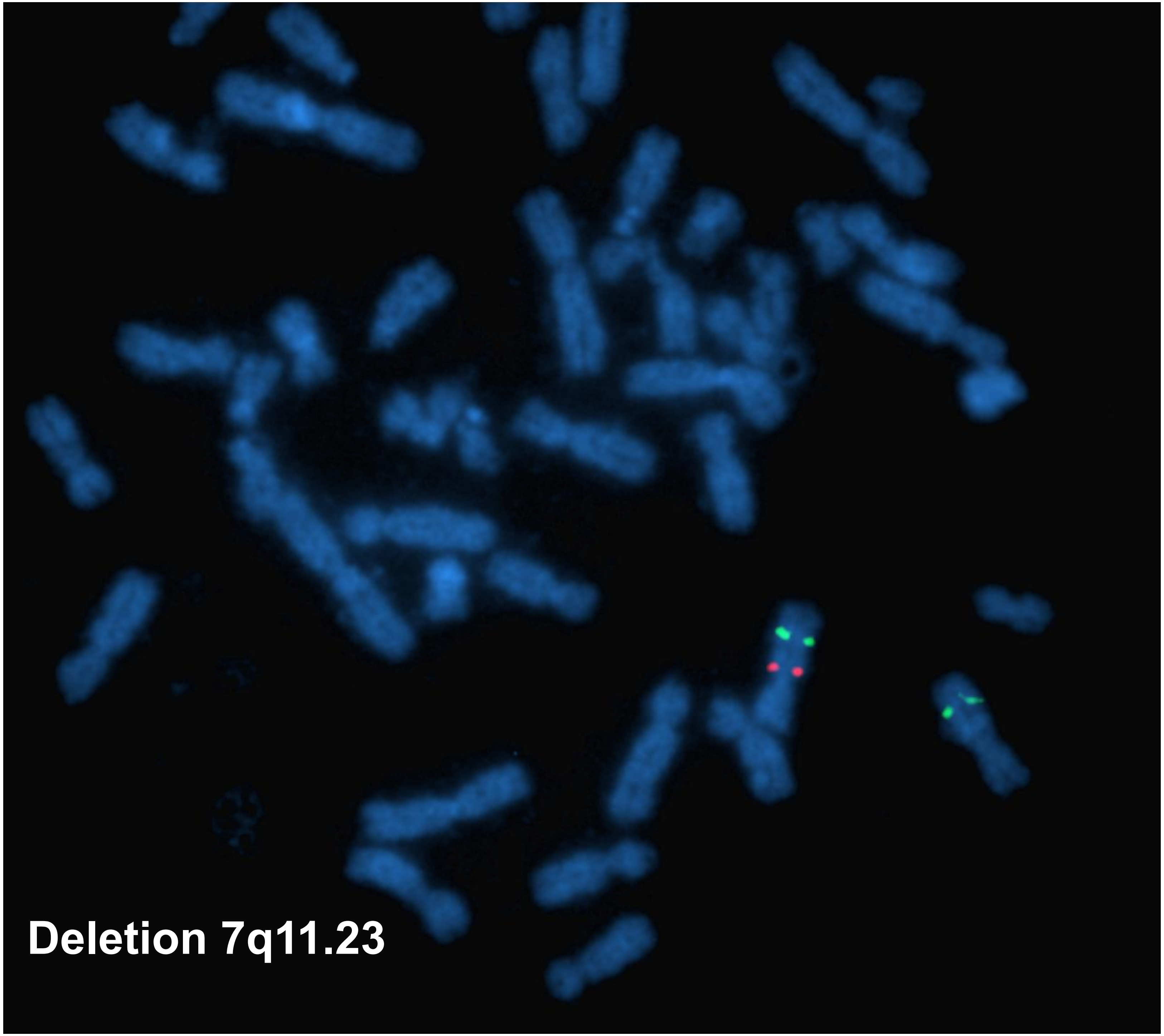
Deletion 7q11.23
